# Performance of 21 HPV vaccination programs implemented in low and middle-income countries, 2009–2013

**DOI:** 10.1186/1471-2458-14-670

**Published:** 2014-06-30

**Authors:** Joël Ladner, Marie-Hélène Besson, Mariana Rodrigues, Etienne Audureau, Joseph Saba

**Affiliations:** 1 Rouen University Hospital, Epidemiology and Public Health Department, Hôpital Charles Nicolle, 1, rue de Germont, 76 031 Rouen, France; 2 Axios International, Paris, France; 3 Hôpital Henri Mondor Hospital, Public Health, Assistance Publique Hôpitaux de Paris, Paris Est University, Créteil, France

**Keywords:** Human papillomavirus vaccine, type 16 L1, 18, Vaccination, Preventive health services, Immunization uterine cervical neoplasms, Developing countries, Vaccines, Program evaluation

## Abstract

**Background:**

Cervical cancer is the third most common cancer in women worldwide, with high incidence in lowest income countries. Vaccination against Human Papilloma Virus (HPV) may help to reduce the incidence of cervical cancer. The aim of the study was to analyze HPV vaccination programs performance implemented in low and middle-income countries.

**Methods:**

The Gardasil Access Program provides HPV vaccine at no cost to help national institutions gain experience implementing HPV vaccination. Data on vaccine delivery model, number of girls vaccinated, number of girls completing the three-dose campaign, duration of vaccination program, community involvement and sensitization strategies were collected from each program upon completion. Vaccine Uptake Rate (VUR) and Vaccine Adherence between the first and third doses (VA) rate were calculated. Multivariate linear regressions analyses were fitted.

**Results:**

Twenty-one programs were included in 14 low and middle-income countries. Managing institutions were non-governmental organizations (NGOs) (n = 8) or Ministries of Health (n = 13). Twelve programs were school-based, five were health clinic-based and four utilized a mixed model. A total of 217,786 girls received a full course of vaccination.

Mean VUR was 88.7% (SD = 10.5) and VA was 90.8% (SD = 7.3). The mean total number of girls vaccinated per program-month was 2,426.8 (SD = 2,826.6) in school model, 335.1 (SD = 202.5) in the health clinic and 544.7 (SD = 369.2) in the mixed models (p = 0.15). Community involvement in the follow-up of girls participating in the vaccination campaign was significantly associated with VUR. Multivariate analyses identified school-based (β = 13.35, p = 0.001) and health clinic (β = 13.51, p = 0.03) models, NGO management (β = 14.58, p < 10^-3^) and duration of program vaccination (β = -1.37, p = 0.03) as significant factors associated with VUR.

**Conclusion:**

School and health clinic-based models appeared as predictive factors for vaccination coverage, as was management by an NGO; program duration could play a role in the program’s effectiveness. Results suggest that HPV vaccine campaigns tailored to meet the needs of communities can be effective. These results may be useful in the development of national HPV vaccination policies in low and middle-income countries.

## Background

Cervical cancer, the third most common cause of cancer in women around the world, is a significant global health challenge, and is the greatest cause of age-weighted years of life lost in the developing world due its high incidence [1,2]. Approximately 291 million women worldwide are estimated to have human papillomavirus (HPV) infection of the cervix [2]. This corresponds to an average prevalence of 10.4% for all women, although the prevalence is higher in women younger than 25 years (16.9%) [3]. HPV is a requirement for developing cervical cancer and is found in 99.7% of cervical cancers diagnosed worldwide [4]. Two HPV serotypes, HPV-16 and HPV-18 are found in nearly 70% of the high-grade cervical lesions that increase a woman’s risk for developing cervical cancer [5].

Since 2006, two prophylactic HPV vaccines have been available, and each has shown > 90% efficacy in preventing HPV type 16- and 18-associated high-grade cervical lesions [6-10]. While both vaccines are being deployed in developed countries, their use of in low and middle-income countries has been limited due to cost and a variety of other factors [11-15]. Recognizing that these factors were impeding the broad use of HPV vaccination in low and middle-income countries, Merck & Co. Inc pledged to donate Gardasil [Human Papillomavirus Quadrivalent (Types 6, 11, 16 and 18) Vaccine, Recombinant] to eligible income countries through the Gardasil Access Program (GAP). Axios Healthcare Development is the recipient of this donation and is responsible for managing GAP. The program was established to enable organizations and institutions in eligible low and middle-income countries to gain operational experience designing and implementing HPV vaccination programs, with the goal of supporting the development of successful child and adolescent immunization models. Most vaccination programs target newborns and young children. In addition, given the substantial morbidity and mortality associated with cervical cancer, effective strategies for its prevention and treatment are critical for improving women’s health throughout the developing world [14]. Pilot programs undertaken in different low and middle-income countries support the use of universal vaccination against HPV as an effective strategy for reducing cervical cancer incidence in these areas [16,17].

Through the provision of vaccines at no cost, GAP aims to foster experience in key implementation areas that are often a challenge in lowest-income countries. These challenges include difficulties in reaching adolescent target populations, resource constraints due to existing needs of routine vaccination campaigns, and selection of optimal vaccination delivery models that most effectively reach the eligible population with the three-dose vaccine series [14,18]. Cultural issues, such as lack of awareness of the connection between HPV infection and cervical cancer, reticence to discuss a sexually transmitted disease, prior negative experience with other vaccine campaigns, safety concerns, and worries about future fertility may also create barriers to deploying HPV vaccination effectively on a broad scale [17-28]. Concerns about public health policy, such as the role of government in deciding who should be vaccinated, may also impact families’ willingness to have their daughters vaccinated [21].

GAP supports programs that are designed and implemented by local and national institutions and organizations. The goal of this approach is to have participating organizations and countries gain experience in the development of relevant and effective strategies for addressing issues that can affect coverage rates, such as infrastructure, culture, and politics. The data gathered from programs implemented under GAP reflect the experiences and lessons learned from multiple countries and represent diverse cultural, political, and health care environments [18,28,29]. These data can be used to guide the development of future national HPV vaccine campaigns in different countries. This approach is consistent with the objectives that the WHO’s Decade of Vaccines Collaboration Research and Development Working Group has outlined in their strategy to address the need for targeted implementation research to improve both coverage of basic vaccines and uptake of new vaccines [30].

While a number of HPV vaccination pilot programs have been undertaken in lowest-income countries, published results from these programs do not typically include information related to program management. Moreover, whereas most published studies describe results from individual programs, GAP provides an opportunity to evaluate a variety of HPV vaccine program types implemented in a number of low and middle-income countries. Consequently, GAP data has the potential to provide important insight into performance factors such as vaccine delivery model, program management and timing, community involvement and sensitization. The aim of this study was to analyze such factors in HPV vaccination programs included in GAP and implemented in low and middle-income countries.

## Methods

### Setting

Organizations and institutions participating in GAP are responsible for covering all other costs associated with their vaccination campaigns, including importation, storage, cold chain management, distribution of vaccine, data collection, and management of the vaccination campaigns (i.e. community outreach and sensitization). GAP encourages participating organizations to adhere to WHO guidelines for HPV vaccination [30]. The WHO recommends that the target age range is 9 or 10 years of age through 13 years of age [30].

### Program inclusion

For inclusion in GAP, interested organizations and institutions completed a detailed application form to describe the characteristics of their institution and their related vaccination experience [31]. Eligible organizations and institutions included both Ministries of Health (MoH) and local non-governmental organizations (NGOs). The application collected information on the managing institution (MoH or NGOs), vaccination implementation plan, estimated target population of girls, logistics, human and financial resources available to support the program, and health services provided by the institution implementing the program. A standardized review form was used to consider the applications; independent experts reviewed the applications and recommended individual programs for inclusion. The GAP Advisory Board reviewed the applications and expert recommendations before issuing a recommendation for final inclusion in GAP.

### Program follow-up

Organizations and institutions participating in GAP are required to submit final program reports once the vaccination program has completed administration of all three doses of vaccine. These reports described final program outcomes, including the number of girls vaccinated per round, the number of girls who received a full course of vaccine, total number of vaccination sites and number of girls vaccinated in each type of delivery site (schools and/or health clinics). The reports also gathered financial data (if available). The reports gathered information related to community involvement actions, communication key messages and methods. The program duration was defined as the time from the date of initial vaccine shipment through the date of delivery of the third vaccine dose (expressed in months).

Three models of vaccine delivery were used: schools, health clinics, and mixed models comprising both schools and health clinic delivery sites. The primary model of vaccination delivery was defined according to the type of site at which > 80% of the targeted population was vaccinated, with mixed models defined as those using both schools and health clinics without either site achieving 80% of target population vaccinations.

### Vaccination indicators

The factors associated with program effectiveness were assessed using two indicators: Vaccine Uptake Rate (VUR) and Vaccine Adherence (VA). The VUR was defined as the number of vaccinated girls that received a full-course of vaccination (three doses) divided by the number of girls targeted. For each program, the number of targeted girls was determined prior to implementation using available population, census and/or school enrollment data, among other sources. The VA was defined as the number of girls receiving a full course of vaccination divided by the number of girls who received a first dose. VA was calculated between doses (D)2 and D1, D3 and D2, and D3 and D1. Adherence between D3 and D1 (VA D3-D1) was used as a metric of program effectiveness. In addition, the speed of each vaccination program was estimated by calculating a VUR per program-month (p-m), which is defined as the number of girls completely vaccinated divided by the number of girls targeted and multiplied by the program duration; speed was expressed in %.

### Cost analyses

The cost analysis was conducted using only those programs that provided comprehensive financial data, including staff costs. Cost data were available for seven of the 21 programs included in this study. Cost categories were not standardized among all programs. Because GAP is a donation program, the cost of vaccine was excluded from the total costs. Two costs measures were used: delivery cost per dose and delivery cost per fully-immunized girl (FIG). Cost per FIG is defined as the cost per dose multiplied by the total number of doses delivered over three vaccination rounds divided by the total number of girls who received all three doses. Program costs were calculated in U.S. dollars (USD).

### Statistical analyses

For continuous variables, data are expressed as mean values with their standard deviation (SD) and median (M). Student t test for parametric quantitative data and Mann–Whitney U test for non-parametric data were used. The Kruskal-Wallis test was used to compare VUR and VA D3-D1 and the three vaccination delivery models. Correlations between the number of vaccination sites, the number of girls vaccinated by program-month, programs duration, and VUR and VA D3-D1 were tested using the Spearman correlation rank (r_s_). Two weighted multiple linear regression analyses were fitted to determine predictive independent factors associated with VUR and VA D3-D1. The two models were weighted on the number of girls vaccinated per program-month. The decision was made to use the same independent variables in the two weighted multiple linear regressions: type of institution (MoH, NGO), vaccination delivery model (school, health clinic and mixed), the number or vaccination sites, and the duration of the program. A significance level of 0.05 was used for all analyses. Statistical analyses were carried out in Statview^®^ 5.0 SAS (SAS Institute Inc.).

### Role of funding source

Merck & Co., Inc. had no role in study design, data collection, data analysis, data interpretation, or manuscript development. All data analyses were conducted with authorization from Merck & Co., Inc. The authors had full access to all data in the study and had final responsibility for the decision to submit for publication. Although Axios Healthcare Development received financial compensation for managing the GAP, the results presented here are a complete and accurate representation of data collected through the study tools.

## Results

A total of 21 HPV vaccination programs in 14 countries were included. Table 1 presents the baseline characteristic of the 21 programs. Thirteen programs (61.9%) were managed by Ministry of Health, and eight programs were managed by an NGO. With respect to vaccine delivery models, 12 programs were school-based, five were health clinic-based, and four programs were mixed models (Table 1).

**Table 1:** Baseline characteristics of the 21 HPV vaccinations programs included in the Gardasil Access Program, 2009–2013

**Program country**	**Managing institution**	**Primary vaccination delivery model**	**Number of girls targeted**	**Number of girls received full vaccine course**	**Total number of vaccination sites**	**Mean total number of girls vaccinated per program-month**	**Vaccine uptake rate %**	**Adherence to vaccination %**		
								**D2-D1**	**D3-D2**	**D3-D1**
Bhutan	MoH	School	3,200	2,721	21	686.1	85.0	96.8	91.3	88.3
Bolivia 1	NGO	School	3,480	3,739	69	732.2	107.4	99.5	96.6	96.2
Bolivia 2	MoH	Mixed	7,500	5,513	19	386.1	73.5	91.6	90.4	82.9
Bolivia 3	NGO	School	30,900	27,597	594	4,039.7	89.3	98.1	98.0	96.2
Bolivia 4	MoH	School	50,000	44,037	2,142	9,650.9	88.1	91.6	95.1	87.1
Cambodia 1	MoH	Mixed	9,600	7,464	10	1,096.2	77.8	91.9	95.5	87.7
Cambodia 2	MoH	Health clinic	2,000	2,027	1	1,096.2	101.4	98.2	97.0	95.3
Cameroon	NGO	Mixed	6,400	5,796	87	348.9	90.6	95.2	88.9	84.7
Georgia	MoH	Health clinic	6,400	4,420	28	502.9	69.1	88.7	102.0	90.4
Haiti	NGO	School	3,300	2,884	162	333.6	87.4	86.8	87.3	75.8
Honduras 1	MoH	School	3,200	3,164	298	523.1	98.9	99.6	98.9	98.5
Honduras 2	NGO	Mixed	1,575	1,472	25	332.5	93.5	99.3	88.9	88.3
Kenya	MoH	Health clinic	3,000	2,500	1	193.3	83.3	95.7	78.1	74.7
Lesotho 1	MoH	School	40,000	33,818	172	257.8	92.6	96.8	95.8	92.7
Lesotho 2	MoH	School	40,100	37,051	436	4,809.6	84.3	94.9	98.5	93.4
Moldova	MoH	School	6,934	6,903	87	3,844.7	99.6	99.9	100	99.9
Nepal 1	NGO	School	3,000	3,164	54	714.6	105.5	99.7	99	98.7
Nepal 2	NGO	School	10,000	9,918	216	351.7	99.2	99.5	99.3	98.8
Tanzania	MoH	School	5,532	4,211	176	2,994.6	76.1	96.1	93.5	89.9
Uganda	NGO	Health clinic	985	937	10	442.2	95.1	94.3	93.5	88.1
Uzbekistan	MoH	Health clinic	8,450	8,450	51	88.6	100.0	100	100	100
Total mean (SD, M)	-	-	245,556	217,786	4,659	1,570.3 (2,336.1, 523.1)	88.7 (10.5, 89.5)	95.9 (3.9, 96.8)	94.6 (5.6, 95.7)	90.8 (7.3, 90.4)

MoH: Ministry of Health. NGO: non-governmental organization. SD: standard deviation. M: median.

Of the 21 programs, seven programs began implementation in 2009 (Bhutan, Bolivia, Cambodia, Haiti, Lesotho, Moldova and Uzbekistan); nine programs in 2010 (Bolivia [2], Cambodia, Cameroon, Georgia, Lesotho, Nepal, Tanzania and Uganda); four programs in 2011 (Bolivia, Honduras, Kenya and Nepal); and one program implemented in 2012 (Honduras). Overall, mean program duration was 10.7 months (SD = 5.0, median M = 9.1). Mean duration for programs conducted by MoH was 10.8 months (SD = 5.7, M = 7.6) and mean duration for programs conducted by NGOs was 10.4 months (SD = 3.7, M = 9.1) (p = 0.88).

The mean VUR was 88.7% (SD = 10.5). The mean VA between D2 and D1, D3 and D2, and D3 and D1 was 95.9% (SD = 3.9), 94.6% (SD = 5.6) and 90.8% (SD = 7.3) respectively (Table 1).

**Figure 1:**
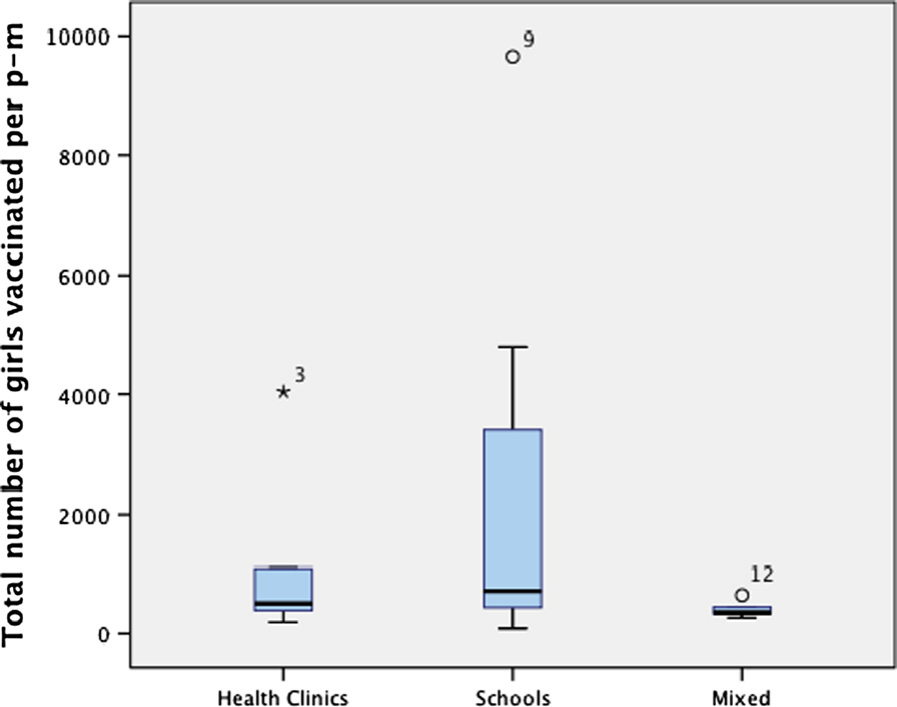
Mean total number of girls vaccinated per program-month (p-m) by vaccine delivery model, Gardasil Access Program, 2009–2013 (N = 21 programs).

The mean total number of girls vaccinated per program-month (p-m) for all programs was 1,570.3 (SD = 2,336.1, M = 523.1) (Table 1). The mean total number of girls vaccinated per p-m by type of institution was 1,811 (SD = 2,765.1, M = 646.7) for MoH programs and 1,178 (SD = 1483.8, M = 513.0) for programs managed by NGOs (p = 0.56). The number of girls vaccinated per p-m by type of program is shown in Figure 1. The mean total number of girls vaccinated per p-m was the highest in the school model, with a mean of 2,426.8 (SD = 2,826.6), 335.1 (SD = 202.5) in mean in the health clinic models and 544.7 (SD = 369.2) in the mixed models (p = 0.15).

Table 2 summarizes the baseline characteristics and program sensitization methods according to VUR and VA D3-D1. As shown, programs managed by an NGO had a higher VUR than programs managed by MoH (96.0% vs. 87.2%, respectively, p = 0.05), whereas the institution type did not impact VA D3-D1. There was no statistically significant difference in VUR and VA D3-D1 based on the health services provided at the institution. The type of delivery model did not have a significant impact on VUR or VA D3-D1 (Table 2).

**Table 2:** Baseline program characteristics and sensitisation methods of programs according to vaccine uptake rate (VUR) and D3-D1 adherence to HPV-vaccination, Gardasil Access Program, 2009–2013 (N = 21 programs)

	**Mean VUR (SD) %**	**p**	Mean D3-D1* adherence to vaccination (SD) %	**p**
**Managing institution**				
MoH (n = 13)	87.2 (10.7)	0.05	90.8 (7.1)	0.99
NGO (n = 8)	96.0 (7.4)		90.8 (8.1)	
**Institution services** Cancer management				
Presence (n = 14)	91.6 (11.3)	0.51	91.7 (7.8)	0.43
Absence (n = 7)	88.4 (8.5)		89.0 (6.5)	
Health care services				
Presence (n = 16)	89.8 (10.6)	0.56	90.2 (7.8)	0.51
Absence (n = 5)	93.0 (10.3)		92.8 (5.5)	
Vaccination services				
Presence (n = 20)	91.1 (10.3)	0.25	90.9 (77.5)	0.87
Absence (n = 1)	80.2 (-)		89.9 (-)	
**Vaccination delivery model**				
HC (n = 5)	89.8 (13.6)		90.1 (9.5)	
Schools (n = 12)	93.1 (8.8)	0.30	92.5 (7.3)	0.52
Mixed (n = 4)	83.8 (9.7)		87.9 (5.1)	
**Community involvement actions**				
Communication key messages definition				
Yes (n = 11)	94.1 (11.4)	0.27	92.5 (7.5)	0.29
No (n = 10)	88.8 (9.2)		89.0 (7.0)	
Girls follow-up				
Yes (n = 13)	95.4 (9.4)	0.05	91.4 (7.5)	0.67
No (n = 8)	86.1 (10.8)		89.9 (7.4)	
Vaccination sessions announcement				
Yes (n = 11)	93.0 (9.4)	0.28	89.3 (9.2)	0.35
No (n = 10)	88.8 (11.5)		92.4 (4.3)	
Girls recruitment				
Yes (n = 14)	89.3 (10.7)	0.44	90.3 (8.3)	0.62
No (n = 7)	93.1 (9.9)		92.0 (5.3)	
Number of community involvements actions				
0 (n = 3)	88.0 (12.1)		90.4 (4.2)	
1-2 (n = 7)	85.7 (10.0)	0.20	89.1 (7.6)	0.72
3-4 (n = 11)	94.4 (9.5)		92.1 (8.0)	
At least one community involvement action				
0 (n = 3)	88.0 (12.1)	0.56	90.4 (4.2)	0.92
> = 1 (n = 18)	92.0 (10.7)		90.9 (7.8)	
**Communication key messages and methods**				
Vaccine administration modalities				
Yes (n = 13)	89.4 (10.2)	0.65	90.3 (8.2)	0.70
No (n = 8)	92.4 (10.2)		91.6 (6.0)	
Vaccine safety and efficacy				
Yes (n = 15)	93.3 (9.1)	0.05	92.3 (6.9)	0.15
No (n = 6)	83.6 (10.8)		87.2 (7.6)	
HPV and link to cancer				
Yes (n = 15)	88.4 (10.3)	0.13	88.8 (7.3)	0.04
No (n = 6)	95.9 (8.9)		95.9 (4.6)	

MoH: Ministry of Health. NGO: non-governmental organization.

*Vaccine adherence between the third and the first dose.

Community involvement in following-up with girls participating in the vaccination campaign was significantly associated with mean VUR, 95.4% (SD = 9.4) versus 86.1% (SD = 10.8) for communities with and without involvement, respectively (p = 0.05) (Table 2). Although VUR increased with the number of community involvement actions, the difference remained insignificant (Table 2). Mean VUR for programs with and without key messages about the safety and efficacy of the vaccine was 93.3% (SD = 9.1) and 83.6% (SD = 10.8), respectively (p = 0.05).

Overall, the mean VUR per p-m was 7.53% (SD = 0.88, M = 7.54). The mean VUR p-m did not differ significantly among the different delivery models: schools (7.73%, SD = 0.78), health clinics (7.48%, SD = 1.13), and mixed models (6.99%, SD = 0.80) (p = 0.35). There was a significant correlation between the VUR p-m and the VA D3-D1 (r_s_ = 0.66, p < 10^-4^), as well as between the total number of girls vaccinated per p-m and the number of vaccination sites (r_s_ = 0.57, p = 0.007). When the correlations were stratified on vaccine delivery models, the three correlations remained positive: health clinic (r_s_ = 0.65, p = 0.27), school (r_s_ = 0.18, p = 0.66), and mixed (rs = 0.72, p = 0.03). There was a negative correlation between the vaccination program duration and VUR (r_s_ = -0.27, p = 0.23), and a significantly negative correlation between duration and VA D1-D3 (r_s_ = -0.48, p = 0.03).

The first weighted multiple linear regression model identified program management by an NGO (p < 10^-3^), school and health clinic delivery model, and duration of the program as significantly independent predictors of VUR (Table 3). In the second weighted multiple linear regression model, no significant association was found between VA D3-D1 and the independent variables fitted in the model, and program duration was the only independent variable that approached statistical significance (p = 0.07) (Table 3).

**Table 3:** Weighted multiple linear regressions* analysis of predictive factors of HPV-vaccination uptake rate (VUR) and D3-D1 vaccination adherence (VA), Gardasil Access Program, 2009–2013 (N = 21 programs)

	**Vaccine uptake rate**			**D3-D1 vaccination adherence**		
	**β coefficient**	**95% CI**	**p**	**β coefficient**	**95% CI**	**p**
Managing institution						
MoH	Ref			Ref		
NGO	14.58	7.64 to 21.52	< 10^-3^	3.90	-2.98 to 10.78	0.24
Number of sites of vaccination	0.01	-0.009 to 0.34	0.23	0.009	-0.01 to 0.03	0.41
Duration of the vaccination program Vaccination delivery model	-1.37	-2.22 to -0.62	0.03	-1.01	-1.98 to 0.45	0.07
Mixed	Ref			Ref		
Health clinic	13.51	1.46 to 25.57	0.03	3.68	-8.28 to15.63	0.66
School	13.35	6.10 to 20.61	0.001	3.00	-4.20 to 10.20	0.89

*Weighted on the number of girls vaccinated by program-month.

MoH: Ministry of Health. NGO: Non-Governmental Organization.

Although comprehensive cost data were not proactively collected from programs participating in GAP, these data were available for seven programs. The mean cost per FIG was USD 8.75 (SD = 4.31, M = 7.10, range = 5.00–17.26) (Table 4). For five programs, the mean cost ranged from USD 5.0 to 7.23, while the two programs with the lowest target populations had substantially higher costs per FIG (USD 11.73 and 17.26). Mean cost per vaccine dose was USD 2.74 (SD = 1.35, M = 2.34, range = 1.38–5.39) (Table 4). There was no significant difference in cost per FIG or per vaccine dose among the three vaccine delivery models, and the cost per FIG was similar in each of the three school-based models included in the analysis (USD 7.02 in Moldova, 7.12 in Lesotho, and 7.23 in Nepal) (data not shown).

**Table 4:** Cost analysis* (in US$) in seven programs included in Gardasil Access Program, 2009–2013 (N = 7 programs)

	**Vaccine delivery model**	**Total costs**	**Cost per FIG**	**Cost per vaccine dose**
Cambodia 1	Mixed	44,157	5.91	1.86
Honduras 2	Mixed	17,269	11.73	3.60
Kenya	Health clinic	12,500	5.00	1.38
Lesotho 2	School	263,815	7.12	2.28
Moldova	School	48,478	7.02	2.34
Nepal 2	School	71,677	7.23	2.39
Uganda	Health Clinic	16,175	17.26	5.39
Total mean (SD, M)	-	474,071	8.75 (4.31, 7.10)	2.74 (1.35, 2.34)

FIG: Fully-immunized Girl.

*Includes staffing costs; excludes vaccine costs.

## Discussion

This study provides insight into factors that impact the outcome of HPV vaccine campaigns in lowest-income countries. Among the key findings of this study are: first, high VUR (mean VUR = 88.7%), and VA D3-D1 (mean VA = 90.8%) were found across the 21 programs assessed; second, school-based vaccine delivery models and program management by an NGO each had a positive and statistically significant impact on VUR; third, duration of the vaccination campaign appears also as a predictive factor for VUR and VA, with increased speed of vaccination predicting higher VUR and VA.

Program management by NGOs was significantly associated with VUR. NGOs are typically smaller and face fewer internal bureaucratic hurdles than a Ministry of Health. Consequently, the finding that NGO management of a program could predict better VUR compared with MoH management may be related to the relative speed with which each type of organization can implement and execute a program, rather than reflecting overall capabilities or competencies of either type of institution. It should also be noted that programs implemented by NGOs have benefited from the authorization and the support of the national health authorities of the country.

Our results found also that program duration is a significant predictor of VUR (p = 0.03) and shows an association with VA (p = 0.07). Shorter duration predicted improved vaccination indicators, suggesting that a longer interval between vaccine shipment and initiation of the third vaccine dose reflects difficulty in vaccinating girls against HPV in a timely manner. A lack of momentum within a given program may lead to loss of interest in completing all three doses of vaccine among girls and their parents. Similarly, a delay in completing all three doses of vaccine may reflect logistical difficulties related to vaccine supplies or personnel. While speed of vaccination has not typically been included in standard efficacy metrics for vaccine programs, these data could suggest that it could be as an aggregate indicator of a well-run program. This result should be confirmed in further studies, and we hope that other investigators will include speed of vaccination in their future studies in order to determine if this metric is a reproducible and reliable predictive factor for program performance. It should be noted that specific program duration was not a requirement imposed on Program grantees by the GAP.

Of the three types of vaccine delivery models assessed in this study, the school-based model was found to be the strongest positive predictive factor of higher VUR. School-based programs likely have the strongest impact on VUR; daily attendance of target girls at school allows them to be vaccinated more quickly than might occur at a health clinic that requires the girl to make a special trip. Given that this finding is based on data from a relatively large number of programs, we believe that this result supports the use of school-based models as a way to optimize delivery of HPV vaccines to school-aged girls. School-based delivery models may be more effective at delivering HPV vaccine because girls between the ages of 9 and 13 years, the target population recommended by the WHO for HPV vaccination, are likely to be present at school in the 12 programs.

The association between school-based models and increased VUR is supported by other studies that demonstrate the relevance of such models in delivering HPV vaccine. A high rate of HPV vaccine coverage (93.2% after three doses) was achieved in Rwanda using a school model [32]. One study found that school-based models have also been effective in demonstration programs in Peru, Uganda, Viet Nam, and India (vaccine coverage ranged from 82.6% to 96.1%) [17], That report suggests that schools may be especially well-suited to reaching girls in the targeted HPV vaccine age range [32]. Additional studies also support the effectiveness of school-based HPV vaccine delivery programs in developing countries [33]. Similarly, a study in Brazil found VURs of 87.5%, 86.3%, and 85.0% at D1, D2 and D3 time respectively, in school-based vaccination delivery programs [34]. In that study, no significant differences in VUR were observed between public and private schools or urban and rural schools [34].

Other studies indicate that school-based models may be more effective than age-based models [32,33,35]. With 84% of children in developing world attending primary school in 2006, school-based models should be considered as an effective method for delivering HPV vaccine to target populations [36]. However, as previously reported, school-based programs may face obstacles if vaccination dates occur on non-school days, and clinic and/or door-to-door follow up may be required in order to complete the three-dose vaccine series [31]. Within the scope of GAP, girls moving away during school breaks or between school years were reported as a key factor that increased the number of girls lost to follow-up. In order to address this issue, it is important that the full vaccination course is administered during the academic school year. This again advocates for the need to carefully monitor the duration of the campaign and to ensure that girls receive all three doses within the recommended vaccine administration schedule that runs for a period of six months. We should also note that while an earlier analysis of only 8 programs participating in GAP found that mixed models were more effective than school-based models, the current analysis contains a larger number of programs, includes a higher number of vaccinated girls, and also utilizes more sophisticated statistical analyses [31].

Community involvement actions appeared to impact the efficacy of the programs, especially the VUR [32]. Programs in which communities were engaged in following-up with girls participating in the vaccination campaign had a higher mean VUR compared with programs that did not engage the community in this activity. This action had no effect on VA, suggesting that enrollment in the vaccine campaign is a bigger hurdle than getting girls who have enrolled to complete the three vaccine doses. There was an interesting trend toward increased VUR with an increase in the number of community involvement actions, suggesting a dose-effect response. However, this trend did not reach statistical significance, which may indicate that the type of activity in which communities are involved may be more important than the overall level of community engagement.

Understanding which aspects of an HPV vaccine campaign are most influenced by community standards, morals, and expectations may help in developing community engagement actions that impact VUR and VA [29]. Community sensitization about the availability and value of vaccinating school-aged girls against HPV may impact vaccine uptake. A study in Brazil found that the method used initially to notify parents about the vaccine had a significant impact on vaccine indicators [34]. That study found that information disseminated by schools was more important than information provided by local media with respect to vaccine uptake [34].

The inclusion of key messages regarding the safety and efficacy of the vaccine had a positive impact on VUR. This finding again suggests that initial enrollment is a greater barrier to completing the three doses of vaccine than is the need to return for repeated vaccine administrations. Key messages that address safety and efficacy at the launch of a vaccine campaign may help to increase enrollment by easing safety concerns and educating girls and their parents about the potential benefits of protection against the long-term consequences of HPV infection. The relatively high rate of VA D3-D1 (90.8%) found in the 21 programs suggests that most girls who participate in the first dose of the campaign remain in the program and complete the vaccine series. These results are consistent with those of a study conducted in Brazil, which found a 97.2% three-dose adherence rate in a school-based HPV vaccine delivery model [34]. This finding is important for those countries interested in implementing an HPV vaccination program to consider [26,34]. Given the availability of census data from the Ministry of Education, and the high density of targeted girls within school populations, school-based delivery models appear to be the optimum approach for reaching girls eligible for vaccination against HPV [33]. Significant effort should be made at the start of any HPV vaccine program to identify all eligible girls and determine the most effective way to include them in the vaccine campaign.

For low and middle-income countries, it is a challenge to conduct effective cost analyses around HPV vaccination. Many countries adapt existing cost models, but these have been developed for high-income countries and may not be relevant [37]. Model predictions using six different vaccination models suggest that vaccination can reduce the incidence of HPV infection and cervical cancer in a cost-effective manner [38]. In these models, factors that influence cost effectiveness are discount rate, duration of vaccine protection, vaccine price, and HPV prevalence. In an effort to provide relevant data that could be used in designing HPV vaccine programs for low- and middle-income countries, we gathered cost data for seven of the GAP programs.

The mean cost per FIG and vaccine dose across the seven programs was estimated to be USD 8.80 and USD 2.70, respectively. Due to the small number programs with cost data available for this study, it was not possible to detect a significant difference in cost per FIG or per vaccine dose among the three models of vaccine delivery in this analysis. However, a study conducted in Tanzania found that the cost per FIG was lower for class-based delivery models compared with age-based delivery in a large schools-based delivery programs including a total of 134 primary schools [39]. Another study in Tanzania found that implementation of a nationwide HPV vaccine program was associated with significant non-vaccine costs, such as financing of pre-introduction activities, development of new delivery infrastructure, and the deployment of new human resources or reallocation of existing personnel [40]. A study that was conducted in Peru, Uganda and Viet Nam and included five HPV vaccination projects found that the cost per vaccine was lower when vaccine delivery was integrated into health services compared with school-based and integrated outreach [41]. Given the importance of building and enhancing the infrastructure for delivering health care to pre-adolescents, investments in non-vaccine costs related to HPV vaccination campaigns could be amortized over a broader array of health services that deliver a variety of interventions to the HPV vaccine target population [33,41].

While our cost data include staffing costs for the programs assessed, they do not provide more detailed information on other non-vaccine costs categories that could substantively impact the implementation or sustainability of the programs [42]. A review of the available data from low- and middle-income countries found that HPV vaccination is cost-effective and potentially cost saving, especially in settings without organized cervical screening programs [43].

Our study has several limitations. First, the 21 programs used various sources of census data that may have been potentially inaccurate to calculate their target populations, which could impact VUR results. Our finding of VUR greater than 100% in three programs may indicate an under-estimation of the target population and/or recruitment of girls from outside of the original target area, suggesting that the methodologies used for determining the target population may be suboptimal. Second, because 21 programs were included in the study, a potential lack of statistical power could be discussed, particularly for the analysis between the sensitization methods used in different programs and VUR and VA results. Consequently, the results from a given model may not accurately reflect the potential performance of that model over an entire population and larger vaccination programs. Third, we have defined program duration as the time from the date of shipment of first vaccine dose to the date of delivery of the third vaccine dose. Although this may not be the most precise measurement of program duration, the definition that we used yielded interesting results and warrants further evaluation. Despite these limitations, this study provides important insight into factors that impact VUR and VA in a very large sample of HPV vaccine programs reaching more than 217,000 girls within a broad range of low and middle-income country contexts.

## Conclusions

The high VUR and VA results across the 21 programs assessed in this study are consistent with previously reported studies from a variety of other HPV vaccine pilot programs [31,32,34,44-47]. A school-based model appeared to be a predictive factor for vaccination coverage, as was management by an NGO. Taken together, these findings suggest that there may not be a “gold standard” vaccination program applicable on a global or even national scale. Rather, tailoring vaccine campaigns to meet the needs, challenges, and cultural priorities of specific regions or communities appears to generate programs with a high rate of success as determined by VUR and VA [48]. The diverse HPV vaccine programs included in this study provide concrete examples of how such programs can be adapted to address local and regional issues and concerns, and provide a useful framework in which countries can consider how to best expand their own HPV vaccination programs based on their individual epidemiological, economic, and health system challenges. The programs included in this study may be used as a starting point for national-scale HPV vaccination programs in their respective countries and related results may also be useful in the development of national HPV vaccination policies in low and middle-income countries.

## Competing interests

Although Axios Healthcare Development received financial compensation from Merck & Co. for the routine management of the Gardasil Access Program (GAP), the results of the 21 programs presented here are a complete and accurate representation of the data collected through the program’s pre-specified follow-up process. Additionally, JL (corresponding author) and EA are not Axios employees and declare that they have no competing interests. These two authors had the primary roles in developing the concept and design of the study, and JL performed the analysis and wrote the manuscript.

## Authors’ contributions

JL conceived the study, wrote the manuscript and performed the analysis. MHB conceived the study, collected data and helped to draft the manuscript. MR helped to draft the manuscript. EA helped to analysis and to draft the manuscript. JS conceived the study and revised the manuscript. All authors read and approved the final manuscript.

## Pre-publication history

The pre-publication history for this paper can be accessed here:

http://www.biomedcentral.com/1471-2458/14/670/prepub
